# Impact of neuromuscular and visuo-motor training on landing biomechanics, functional performance, and psychological factors in female athletes with unilateral anterior cruciate ligament reconstruction: a simple parallel-group randomized controlled trial

**DOI:** 10.1186/s13018-026-07141-3

**Published:** 2026-07-31

**Authors:** Ahmad Kazemi Pakdel, Fereshteh Kazemi Pakdel, Nader Farahpour

**Affiliations:** 1 Corrective Exercises and Sports Injury Department, Faculty of Physical Education & Sport Sciences, University of Guilan, Rasht, 4199613776 Iran; 2 Faculty of Physical Education and Sport Science, Bu-Ali Sina University, Hamedan, 6517838695 Iran

**Keywords:** Anterior cruciate ligament, Biomechanics, Neuromuscular training, Rehabilitation visuo-motor training

## Abstract

**Purpose:**

This study aims to compare the effects of neuromuscular training (NMT) and visuo-motor training (VMT) on the biomechanics of the landing task, functional performance, and psychological factors in female athletes with a history of unilateral anterior cruciate ligament reconstruction (ACLR).

**Materials and methods:**

A total of 45 competitive female athletes (age: 27.83 ± 4.44; height: 167.58 ± 7.52 ± ; BMI: 23.4 ± 1.93) with unilateral ACLR were recruited. Participants were randomly assigned to one of three groups: the NMT group (n = 15), the VMT group (n = 15), and a control group (n = 15). Using a video camera and a force plate system, Kinematic and kinetic data were evaluated during a landing task. Functional performance was assessed through the triple hop test and the Y-Balance Test (YBT). Additionally, kinesiophobia was measured using the Short Anterior Cruciate Ligament-Return to Sport after Injury scale (ACL-RSI). Data analysis was conducted in SPSS 28.0, employing a repeated-measures ANOVA (3 groups × 2 time points).

**Results:**

Both the NMT and VMT showed significant improvements in peak hip flexion (p = 0.02, Effect size (EF) = 1.13 for NMT, p = 0.02, EF = 1.22 for VMT), peak knee flexion (p = 0.02, EF = 1.21 for NMT, p = 0.02, EF = 1.23 for VMT), peak dorsiflexion (p = 0.001, EF = 1.56 for NMT, p = 0.001, EF = 1.53 for VMT), peak knee valgus (p = 0.03, EF = 0.98, for NMT, p = 0.04, EF = 1.01 for VMT), GRFv (p = 0.001, EF = 1.42 for NMT, p = 0.001, EF = 1.39 for VMT), YBT (p = 0.02, EF = 1.11 for NMT, p = 0.03, EF = 1.12 for VMT), triple hop test (p = 0.02, EF = 1.15 for NMT, p = 0.02, EF = 1.16 for VMT), and ACL-RSI scale (p = 0.001, EF = 1.62 for NMT, p = 0.001, EF = 1.63 for VMT). Also, there was no significant difference between the two NMT and VMT groups in all measured variables (p > 0.05).

**Conclusion:**

Both interventions resulted in significant improvements in the lower limbs’ angle, GRF_V_, and psychological factors in female athletes with unilateral ACLR. While both NMT and VMT are effective rehabilitation approaches, providing clinicians with distinct options, it is important to acknowledge key methodological limitations, including the small sample size and short follow-up duration.

**Supplementary Information:**

The online version contains supplementary material available at https://doi.org/10.1186/s13018-026-07141-3.

## Introduction

Anterior cruciate ligament (ACL) injuries predominantly occur during athletic activities characterized by abrupt stops, directional changes, jumping, or landing [1]. Following such injuries, athletes experience significant biomechanical asymmetries across multiple planes, which can lead to the loss of an entire season of their sport [2]. The recovery process is often lengthy and challenging, and there is a potential for decline in athletic performance upon returning to sport [3, 4]. Furthermore, previous studies indicate that athletes experiencing negative psychological factors, such as anxiety, mood disturbances, depression, and reduced athletic identity, are less likely to successfully return to sports (RTS) [5, 6]. Returning to sport too soon, before achieving psychological readiness—characterized by the right mindset, emotional stability, and confidence can lead to anxiety, increased reinjury risk, injuries to other body parts, and decreased performance [7, 8]. Consequently, there is an increasing emphasis on understanding the psychological recovery process following serious injuries.

For those who do return to their previous activity levels, the risk of sustaining a second ACL injury, either in the same knee or the opposite knee, can be as high as 29% [9]. This elevated risk is often attributed to diminished muscle strength and function, which contribute to altered jump-landing biomechanics [10]. Key biomechanical factors associated with the risk of a second ACL injury include increased vertical ground reaction force (GRF_V_), reduced hip and knee flexion, and heightened knee abduction and internal rotation [11]. These factors collectively result in a dynamic alignment known as "knee valgus." Targeted training interventions aimed at addressing these biomechanical issues may significantly mitigate the risk of subsequent ACL injuries before unrestricted participation in sports [12].

The literature has shown certain benefits and a reduced risk of ACL injury when using ACL injury prevention programs [13, 14]. One of the most popular prevention programs for ACL injuries is neuromuscular training (NMT). NMT programs are crucial for preventing ACL injuries by including lower extremity strengthening, plyometrics, balance training, and movement retraining [15]. They effectively reduce ACL injury rates by improving landing mechanics, mitigating biomechanical risk factors, and enhancing muscle activation, particularly in the hamstrings [16]. For ACL reconstruction (ACLR) recovery, these programs improve knee pain and function more than traditional strength training, while also reducing anterior tibial displacement and enhancing joint stability [17].

Despite the positive effects of neuromuscular training and other exercises on reducing ACL re-injury rates [18], challenges remain in ensuring consistent implementation and adherence to these training programs among athletes. Research indicates that approximately two-thirds of athletes do not return to their pre-injury performance level one year post-ACL reconstruction [19]. Longitudinal studies show that only 65% of athletes achieve their prior competitive level at an average follow-up of nearly 3.5 years, despite having restored normal knee function [20]. Furthermore, seven years after surgery, merely 36% continue to engage in their original sports [21]. The field of biomechanics has undergone a transformative evolution, driven by rapid advancements in both hardware and software technologies. These innovations have not only enhanced our understanding of human movement but have also bridged the gap between research and practical applications in sports medicine, performance optimization, and injury rehabilitation [22].

Asymmetries in leg coordination are frequently observed after ACL injuries or ACLR, and current rehabilitation programs often fail to effectively address these issues, leading to increased risks of secondary injuries and early knee osteoarthritis [23]. Traditional rehabilitation methods, which rely on predefined movement patterns, do not restore normal motor function for all patients [24]. In contrast, newer approaches focus on self-organization and individualized movements, incorporating principles of motor learning and introducing variability to enhance implicit learning, such as differential learning (DL) [25]. These methods aim to reduce working memory dominance and frontal lobe activation, with DL specifically overloading working memory to trigger qualitative changes [26]. Additionally, visuo-motor training (VMT) is suggested to address visual perturbations in ACLR rehabilitation [27].

VMT is an innovative approach in ACL rehabilitation that emphasizes "more accurate feedforward motor control" by focusing on visuo-motor information during practice [28]. It aims to enhance recovery through sensory reweighting, reducing post-injury reliance on visual information and promoting greater dependence on proprioceptive inputs from the joint capsule, ligaments, and muscle spindles [27]. A key aspect of VMT is visual obstruction training, which sensitizes proprioceptive and vestibular inputs during standard rehabilitation exercises [28]. By decreasing reliance on vision, VMT shifts neural processing towards proprioception, improving visual processing efficiency [29]. The theoretical basis for VMT is supported by research demonstrating neurophysiological changes in athletes following ACLR. These studies indicate that modifications in visual input can enhance sensorimotor processing. Improved sensorimotor processing is essential for effective movement planning and neuromuscular control. Additionally, these changes promote better cortical top-down motor control strategies, leading to improved functional outcomes [28]. VMT offers a unique approach to ACL rehabilitation by emphasizing proprioceptive inputs over visual cues, which may enhance psychological factors such as confidence and reduce kinesiophobia [28]. Kinesiophobia, or fear of movement, often arises after an injury due to concerns about re-injury or pain. By integrating VMT, patients gradually learn to rely less on visual feedback, helping to diminish the fear associated with movement and encouraging more confident engagement in physical activity [30]. As patients undergo visual obstruction training, they become more attuned to their body’s position and movements, fostering a sense of control and mastery over their physical capabilities, which improves self-efficacy and reduces anxiety related to movement [28].

In our study, we aim to compare neuromuscular training and VMT due to their distinct yet complementary theoretical approaches in addressing the multifaceted challenges faced by athletes recovering from ACL injuries [27]. While neuromuscular training focuses on enhancing lower extremity strength, balance, and movement mechanics to reduce biomechanical asymmetries and reinjury risk, VMT targets the integration of visual and proprioceptive inputs to improve motor control and sensory processing [15]. We hypothesize that VMT may be superior in improving reaction times and psychological factors, while NMT may be more effective for enhancing strength outcomes. This comparison will help clarify the specific benefits of each training modality for athletes in recovery.

Given that ACL injuries often result in significant biomechanical deficits and psychological barriers it is crucial to understand whether these fundamentally different training modalities produce similar or divergent effects on recovery. By comparing their impacts on landing biomechanics, functional performance, and psychological factors, we hope to determine their relative efficacy and identify comprehensive rehabilitation strategies that can enhance recovery outcomes and support a safe return to athletic activity. This comparison is vital for expanding the repertoire of evidence-based protocols that address both the physical and mental aspects of recovery, and for investigating whether different neural pathways can be leveraged to achieve similar functional gains, which is the focus of our investigation into the impact of neuromuscular and visuo-motor training on these critical factors in female athletes with unilateral ACL reconstruction.

## Methods

### Participants

Forty-five competitive female athletes from handball, volleyball, and basketball, all of whom had undergone primary ACLR and completed conventional post-operative rehabilitation, voluntarily enrolled in the study. A power analysis was conducted to determine the sufficient sample size of each group. The sample size was calculated based on a previous study by Gholami, Letafatkar (28) with an alpha level of 0.05, effect size of 0.4 and an actual power (1-beta) of 0.80. The analysis (G × Power, Version 3.1.9.2, University of Kiel, Germany) revealed that a sample size of *n* = 12 would be sufficient for each group to find significant effects between variables. To account for potential participant dropouts, the number of participants was increased by 10% for each group, ensuring adequate power for the study. A blinded investigator performed the randomization process, assigning eligible participants to one of three groups: the NMT group (n = 15), the VMT group (n = 15), and a control group (n = 15) (see Table 1).

**Table 1:** Baseline measurements (mean ± SD)

Characteristic		NMT group (n = 15)			VMT group (n = 15)			Control group (n = 15)			p-value
		mean	±	SD	mean	±	SD	mean	±	SD	
Age	(years)	27.11	±	4.29	28.73	±	4.19	27.67	±	4.86	0.89
Height	(cm)	166.7	±	7.6	168.3	±	7.11	167.76	±	7.86	0.76
Body mass	(kg)	64.76	±	8.95	63.89	±	8.11	63.12	±	8.17	0.69
BMI	(kg/m^2^)	23.9	±	1.98	23.2	±	1.73	23.1	±	2.1	0.89

^*^Significance level was considered p ≥ 0.05

BMI: Body mass index; NMT: Neuromuscular training; VMT: Visual-Motor Training

Inclusion criteria were as follows: participants must have undergone unilateral hamstring tendon autograft ACLR performed by the same surgeon 6 to 12 months before participation. Athletes were required to be pain-free with no effusion, demonstrate full active knee range of motion, and be medically cleared for sports participation. Functional criteria included a quadriceps strength index of ≥ 80% [31], limb symmetry confirmed by handheld dynamometry, and successful completion of all hop tests (≥ 80% limb symmetry index and pain-free [32]. Exclusion criteria included any concomitant injury to another knee structure (e.g., medial collateral ligament, meniscus) or experiencing a post-operative re-injury [33]. All participants provided written informed consent prior to the study.

### Study design

This study employed a simple parallel-group, randomized controlled trial design with a convenience sampling method, a limitation that may reduce the external validity and generalizability of the findings. The study followed the CONSORT 2025 guidelines for reporting randomized trials [34]. Participants were randomly assigned to one of three groups: two experimental groups or one control group, using computer-generated random numbers. The randomization was performed using a computer-generated block randomization table. Randomization was carried out by an independent investigator who was not acquainted with the testing protocol, utilizing a random number table to ensure unbiased group assignment. To further reduce the risk of bias, group allocation was concealed within opaque envelopes until after participants were enrolled in the study. For the purposes of blinding, one researcher administered both the pre-test and post-test assessments, while a different researcher implemented the exercise programs, thereby maintaining the integrity of the study's design. The study lasted 11 weeks, structured as follows: week 1 served as a familiarization period; week 2 was designated for pretesting; weeks 3 to 10 constituted the training period; and week 11 was the post-test period. Participants were invited to the gym for reassessment of their performance. Two weeks prior to the start of the training, a researcher communicated with the participants to provide necessary information. To standardize the training procedures, three weekly exercise sessions were scheduled to allow participants to become acquainted with the training protocol. During these sessions, participants were instructed on how to perform the exercises correctly, with all instructions provided through comprehensive communication and demonstrations. The examiners for the biomechanics and functional performance tests were blinded to group allocation.

This study was conducted after obtaining approval from the institutional ethics committee under the code IR.IAU.SARI.REC.1404.272, and all procedures were carried out in accordance with relevant guidelines and regulations.

### Procedures

The participants underwent testing over two days, which included both pre-test and post-test assessments. To minimize the impact of circadian variations on the test results, all assessments were conducted at the same time of day (between 10 AM and 12 PM), during the same training hours, and on the same day of the week as the pre-test. Additionally, participants were instructed to avoid any physical activity or practice before the tests began. This standardized protocol helped ensure reliable and comparable results. The current study adheres to the findings presented by Lu, Xu [35], which indicate that different types and intensities of exercise prescriptions can significantly affect physiological recovery in postoperative populations. It highlights the necessity of incorporating novel visuoaudio–motor training as a potential alternative to traditional neuromuscular training in the rehabilitation of ACLR. This approach underscores the importance of tailored exercise prescriptions to optimize recovery outcomes for patients.

### Measurements

Height was measured using a wall-mounted stadiometer (Seca 222, Terre Haute, IN) and recorded to the nearest 0.5 cm [36]. A digital scale (Tanita, BC-418MA, Tokyo, Japan) was also used for calculating body mass index (kg/m^2^) [36].

#### Biomechanics testing

Kinematic data were captured using two CASIO Exilim EX-ZR700 high-definition video cameras configured as follows: resolution of 1280 × 720 pixels; frame rate of 120 Hz [37]; focal length of 52 mm; sensitivity set to ISO 400; aperture at f/2.7; and shutter speed adjusted accordingly. The cameras were positioned at a height of 0.98 m above the ground, recording the landing task at 30 frames per second. They were situated on either side of the landing path; each was positioned two meters away from the point of landing. GRF_V_ data were recorded at 1500 Hz via a model BA 9286 Kistler force plate. Retroreflective markers were strategically placed on specific anatomical landmarks of the pelvis and lower limbs, following the Plug-in-Gait lower body marker set. These markers included the right and left anterior superior iliac spines, right and left posterior superior iliac spines, lower lateral surfaces of the right and left thighs (aligned with the hip and knee joint markers), right and left lateral epicondyles of the femur, lower lateral surfaces of the right and left tibias (aligned with the knee and ankle joint markers), right and left lateral malleoli, the superior proximal ends of the second metatarsals of both feet, and the posterior aspect of the Achilles tendons at the same height as the second metatarsal markers. For the single-legged drop landing task, participants began from a single-leg standing position on a 25 cm high platform adjacent to the force plate. The athletes landed on the force plate with the limb corresponding to the ACLR and then jumped upward as high as possible. Each athlete practiced the landing task four times, with three trials recorded for analysis. The average of these three landings was used for statistical evaluation, and no feedback was provided during data collection. GRF_V_ data from the single-leg landing trials were processed using a fourth-order, zero-lag recursive Butterworth filter, with a cutoff frequency of 500 Hz for force data [38]. Two-dimensional joint angles for the hip, knee, and ankle were computed using an XYZ Cardan sequence. These joint angles represented the orientation of the distal segment's local coordinate system relative to the proximal segment’s coordinate system. In our study, we employed the Plug-in-Gait marker set in conjunction with the two-dimensional analysis. This approach allowed us to leverage the detailed anatomical reference provided by the marker set while focusing on the two-dimensional joint angle calculations. GRF_V_ variables were analyzed during the first 100 ms after initial contact with the force plate. Loading rates were determined by dividing the peak vertical ground reaction force (GRF_V_) by the time taken to reach peak force. All GRF_V_ variables were normalized to body mass (e.g., N/kg) as appropriate. Data processing was conducted using custom MATLAB scripts (The MathWorks, Inc., Natick, MA, USA) to extract peak GRF_V_ during the initial landing phase of the single-legged landing task [17].

#### Functional performance testing

Triple Hop Test. Functional performance was assessed during three trials of a single-leg horizontal triple hop test. Prior to the actual testing, the athletes performed practice trials to familiarize themselves with the task. A standard tape measure, positioned perpendicular to the starting line, was used to record the distance hopped. The single-leg triple hop task is a widely recognized and reliable functional performance assessment for athletes who have undergone ACLR, with an intraclass correlation coefficient (ICC) ranging from 0.93 to 0.98. [39]

Y-Balance Test (YBT). To evaluate lower-body balance performance, the YBT was conducted according to the methodology outlined by Ko, Wikstrom [40]. The composite score was calculated by analyzing the scores from three directional reaches anterior, posteromedial, and posterolateral integrating them into a single measure of balance performance. YBT scores were normalized by dividing the reached distance by the participant’s leg length and multiplying by 100 to express the results as a percentage. Leg length was measured in centimeters from the anterior superior iliac spine to the center of the ipsilateral medial malleolus, with the participants in a supine position.

#### Kinesiophobia testing

Kinesiophobia was evaluated using the Iranian version of the short form of the Short Anterior Cruciate Ligament-Return to Sport after Injury scale (ACL-RSI). The original short version consists of six items assessed via a 10-cm visual analog scale, which ranges from 0 to 100 in 10-point increments. This instrument includes three subdomains: (1) emotions (3 items), (2) confidence in performance (2 items), and (3) risk appraisal (1 item). In a subsequent adaptation by the same research team, the visual analog scales were replaced with 11-point Likert scales presented in boxes, maintaining 10-point increments from 0 to 100. The total score is calculated as a percentage of the total scores across the six items, with higher scores indicating greater psychological readiness to return to sport [41]. The reliability and validity of the short version of the ACL-RSI have been established in Persian, with an intraclass correlation coefficient (ICC) of 0.92 [42].

### Interventions

#### Neuromuscular training program (NMT)

After the initial baseline assessments, athletes in the NMT group engaged in an 8-week progressive NMT regimen, supervised by a qualified athletic trainer with extensive experience. This program aimed to enhance lower-limb strength, control, power, balance, and landing techniques. The protocol’s effectiveness in increasing hip strength and hop distance, while decreasing risky landing mechanics, has been documented previously [43]. The training protocol consisted of 22 sessions over 8 weeks, with a frequency of 3 sessions per week for the first 6 weeks, tapering to 2 sessions per week for the final 2 weeks. The training protocol comprised eight specific exercises: double-leg squats, walking lunges, single-leg squats, double-leg drop jumps, single-leg stances on unstable surfaces, single-leg countermovement jumps, horizontal bounds, and single-leg standing long jumps, all performed using body weight as resistance. A detailed weekly breakdown, specifying the exercises, along with their corresponding sets, repetitions, and duration, is provided in Table 2. The proposed program aligns with current guidelines for primary prevention of ACL injuries [44].

**Table 2:** Neuromuscular Training Program Details

Exercise	Wk 1	Wk 2	Wk 3	Wk 4	Wk 5	Wk 6	Wk 7	Wk 8
Double-leg squats	3 × 6	3 × 6	3 × 6	4 × 8	4 × 8	4 × 12	3 × 6	4 × 8
Walking lunges	3 × 6	3 × 6	3 × 6	4 × 8	4 × 10	4 × 12	3 × 8	5 × 10
Single-leg squats	3 × 6	3 × 6	3 × 6	3 × 8	4 × 8	4 × 10	3 × 8	3 × 8
Double-leg drop jumps	3 × 6	3 × 6	3 × 6	4 × 8	5 × 8	5 × 10	4 × 8	4 × 8
Single-leg stance, unstable surface	3 × 6	3 × 6	3 × 6	3 × 30 s	3 × 30 s	4 × 30 s	4 × 30 s	3 × 30 s
Single-leg countermovement jumps	3 × 6	3 × 6	3 × 6	4 × 8	4 × 10	3 × 8	3 × 8	3 × 8
Horizontal bounds	3 × 6	3 × 6	3 × 6	4 × 8	5 × 10	3 × 8	3 × 8	3 × 8
Single-leg standing long jumps	3 × 6	3 × 6	3 × 6	4 × 8	5 × 8	3 × 8	3 × 8	3 × 8

^a^ Sets and Repetitions/Time: The table outlines the sets and repetitions or time for each exercise across the 8-week period

^b^ Wk = Week

^c^ Rest Period: Athletes were given 30–60 s of rest between sets

#### Visual-Motor Training (VMT)

Athletes in the VMT group participated in an 8-week training program, which included three sessions per week. Each training session lasted approximately 25 to 30 min. The VMT protocols were specifically designed for stroboscopic intervention and were incorporated into the regular training regimen (see Table 3) [45]. These protocols included various tasks such as a Tap-test, agility ladder drills, single-leg stances on foam, vertical jumps, and squat jumps. An error scoring system for evaluating performance while using stereoscopic glasses (SG) is outlined in Table 4. In the VMT program, athletes executed the protocol under stroboscopic conditions. Each week, the settings of the shutter glasses—specifically the frequency (in Hz) and duty cycle (in %)—were modified to accommodate anticipated enhancements in visuomotor performance and to prevent adaptation to any particular setting [45]. Furthermore, athletes were instructed to avoid looking at their feet and to concentrate on executing the movements with proper form.

**Table 3:** Visual-motor training exercises

Exercise	Visual Cues/Modifications
Tap-test	Run 10 m to tap a cone Cut right or left for 5 m to tap another cone Cut to the opposite direction for 10 m to tap the third cone Return to the center by cutting 5 m to tap the first cone, then run 10 m back to the start position (forming a "T") Acoustic Cue: Clinician calls out “Left” or “Right” to indicate cutting direction, increasing cognitive demands
Agility Ladder Drills	Match specific foot-placement patterns within the agility ladder
Single-leg Deadlifts	Gently place a small object on the ground next to a cone target To increase difficulty, multiple cones can be placed at different angles, and the clinician may call out “Left,” “Center,” or “Right” to vary the task order and difficulty
Single-leg Stance (on foam)	Hold a lightweight bar with an outstretched arm and focus on keeping it horizontal
Vertical Jumps	Use the VERTEC to assess maximum vertical jump height While aiming to hit a target equal to 80% of their maximal jump height, clinicians may call out “Left” or “Right” during the jump to signal unilateral landing Spontaneous cuing creates an unanticipated landing task, increasing injury-risk biomechanics
Squat Jumps	Place four cones around the participant at 0, 90, 180, and 270-degree positions Number each cone one through four Clinician rapidly calls out cues to specify which cone the athlete should face after each jump squat Increase difficulty by introducing more cones or increasing the rapidity of cuing

Clinicians should ensure that athletes can successfully perform all exercises before introducing stereoscopic glasses. Once athletes demonstrate proficiency, clinicians can begin using the glasses at the easiest difficulty level, which features the highest frequency of fluctuation between transparent and opaque states. As athletes show improvement in their performance, clinicians can progressively increase the difficulty of the stereoscopic glasses to enhance the visual-cognitive demands of the training

**Table 4:** Error Scoring System Used to Assess Behavioral Performance

Exercise	Error Count
T-test	Miss a cone
	Cut to the wrong direction
Agility Ladder Drills	Hit the ladder
	Incorrect foot placement
Single-leg Deadlifts	Opposite foot touches ground
	Either hand touches ground
	Object placed in wrong location
Single-leg Stance (on foam)	Opposite foot touches ground
	Either hand touches ground
Vertical Jumps	Miss the target
	Land on wrong foot
Squat Jumps	Land facing wrong orientation

#### Control group

Participants in the control group engaged in matched, non-specific physical activities, including light aerobic exercise and stretching, as well as other standard return-to-sport activities. This approach was implemented to control for the effects of training attention and total time commitment, ensuring that the control group did not experience a detraining stimulus that could artificially inflate the relative success of the intervention groups.

### Statistical Analysis

Data normality was assessed using the Shapiro–Wilk Normality test. The results are presented as mean ± standard deviation (SD). Baseline characteristics of the participants were analyzed using a One-way ANOVA. Data analysis was conducted in SPSS 28.0, employing a repeated-measures ANOVA (3 groups × 2 time points) [46]. Mauchly’s test of sphericity was used to evaluate the sphericity assumption, and violations were corrected using the Greenhouse–Geisser (GG) adjustment. When a significant main effect was found (p < 0.05), a Bonferroni post hoc test was performed to identify specific differences. The effects of training were quantified using effect sizes (ES), calculated with Cohen’s d [47]. The magnitude of the effect sizes was interpreted as: < 0.2 (trivial), 0.2–0.6 (small), 0.6–1.2 (moderate), 1.2–2.0 (large), 2.0–4.0 (very large), and > 4.0 (nearly perfect) [48]. The effect sizes are reported with 95% confidence intervals (CI).

## Results

**Fig. 1:**
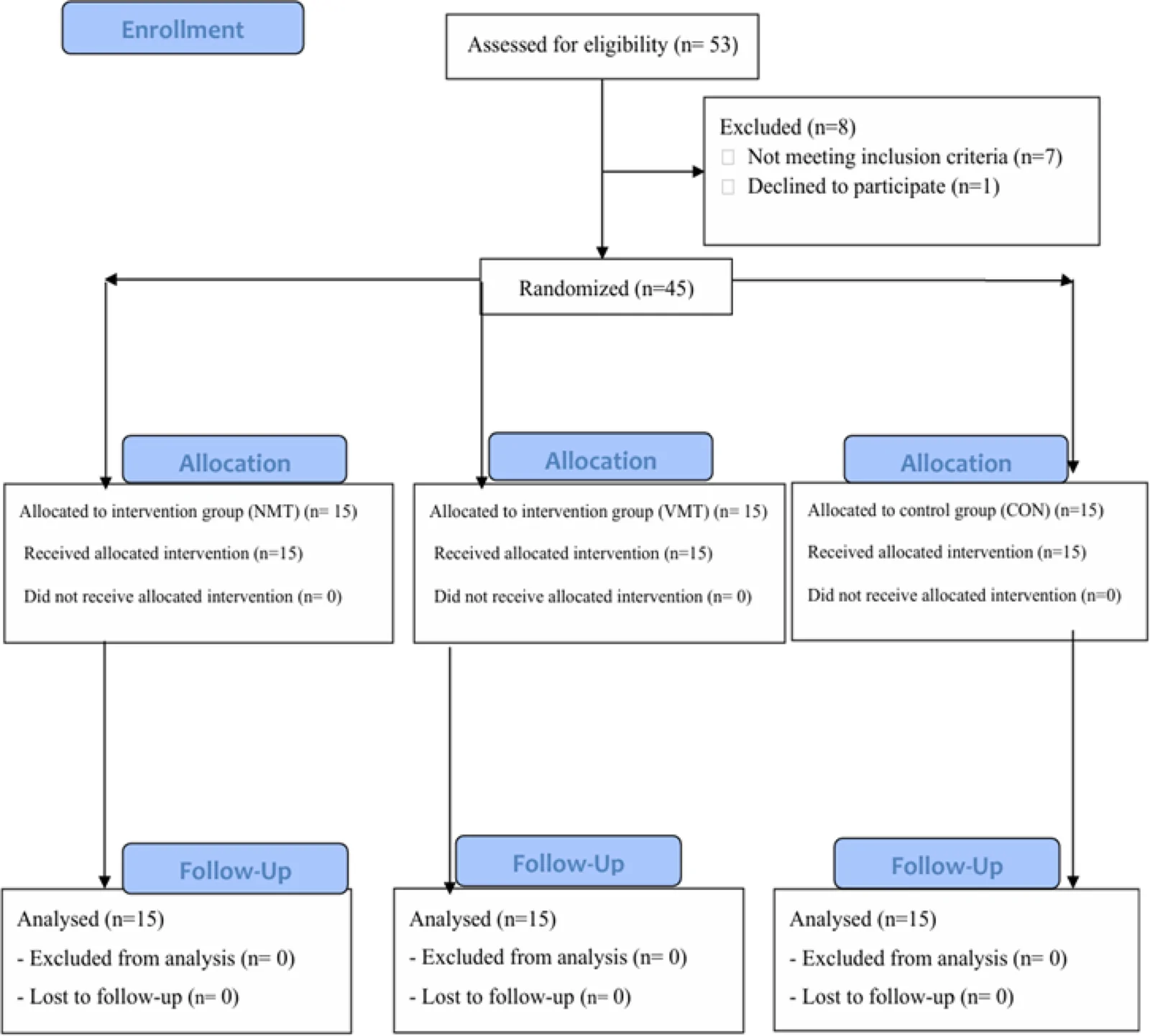
Flowchart of eligibility, inclusion and exclusion criteria, and analysis; NMT: Neuromuscular training; VMT: Visual-Motor Training

In the initial phase of the study, 53 competitive female athletes with ACLR were recruited. Following the application of inclusion and exclusion criteria, 45 participants were selected and randomly assigned to three groups, with 15 individuals in each group. To be included in the final analysis, participants had to complete all training sessions and attend all assessment sessions. All participants met the study requirements, resulting in no dropouts; all 45 participants were retained for the final analysis. This impressive retention rate can be attributed to effective compliance strategies, including regular follow-up communications, motivation through goal-setting sessions, and providing participants with clear instructions and support throughout the 11-week study. These strategies contributed to maintaining high adherence rates among participants (see Fig. 1).

At baseline, no significant differences were detected among the groups for the dependent variables (p > 0.05). Additionally, no significant changes were observed in the control group (CON) across all variables following the training period (p > 0.05).

The repeated-measure ANOVA revealed a significant group-by-time interaction for all primary outcome measures: peak hip flexion (F = 4.63, p = 0.014), peak knee flexion (F = 5.3, p = 0.001), peak dorsiflexion (F = 4.35, p = 0.017), peak knee valgus (F = 5.23, p = 0.001), peak GRF_V_ (F = 4.9, p = 0.012), YBT (F = 5.1, p = 0.011), triple hop test (F = 6.2, p = 0.001), and ACL-RSI scale (F = 5.42, p = 0.001).

Post-hoc analysis demonstrated that both the NMT and VMT groups showed significant improvements from pre-test to post-test. They exhibited increased peak hip flexion (p = 0.02 for NMT, p = 0.02 for VMT), peak knee flexion (p = 0.02 for NMT, p = 0.02 for VMT), peak dorsiflexion (p = 0.001 for NMT, p = 0.001 for VMT), GRF_V_ (p = 0.001 for NMT, p = 0.001 for VMT), and YBT scores (p = 0.02 for NMT, p = 0.03 for VMT), triple hop distance (p = 0.02 for NMT, p = 0.02 for VMT), and ACL-RSI scale scores (p = 0.001 for NMT, p = 0.001 for VMT) scores. Furthermore, both groups showed a significant reduction in peak knee valgus (p = 0.03 for NMT, p = 0.04 for VMT) and peak GRF_V_ (p = 0.04 for NMT, p = 0.001 for VMT). However, there was no significant difference in post-test outcomes between the NMT and VMT groups for all measured variables (p > 0.05). The results indicate that both interventions showed improvements and that no statistically significant between-group differences were observed. The effect sizes were found to be similar, further indicating comparable effectiveness. Detailed information regarding these effect sizes is provided in Table 5. Additionally, it is important to note the overlap of 95% confidence intervals, which further supports the conclusion of "no difference" between the two training modalities. (Table 5).

**Table 5:** Significant and effect size for the variables in the groups [n = 45]

Variables	Groups	Time point values (mean ± SD)		Statistic	Within-group Effect size (95% CI) and p-Value	between-group NMT–VMT mean differences with 95% confidence	
		Pre	Post		Pre to post ES		p-value
Peak hip flexion	NMT	52.86 ± 8.86	59.44 ± 9.12*	G = 0.001	1.13 (0.36 to 1.92) d	1.42 (0.17 to 1.89)	0.02
	VMT	53.76 ± 9.11	60.86 ± 8.11*	T = 0.001	1.22 (0.47 to 1.83) d		0.02
	CON	51.89 ± 9.76	50.86 ± 9.37	G × T = 0.001	-		
Peak knee flexion	NMT	29.43 ± 6.19	35.11 ± 5.76*	G = 0.001	1.21 (0.38 to 1.94) d	1.08 (0.22 to 1.37)	0.02
	VMT	30.35 ± 6.73	36.19 ± 5.73*	T = 0.02	1.23 (0.32 to 1.86) d		0.02
	CON	29.43 ± 5.38	35.12 ± 5.11	G × T = 0.001	-		
Peak dorsiflexion	NMT	16.86 ± 3.49	22.43 ± 3.86*	G = 0.03	1.56 (0.16 to 1.76) d	0.68 (0.43 to 1.46)	0.001
	VMT	16.29 ± 3.46	23.11 ± 3.43*	T = 0.02	1.53 (0.18 to 1.75) d		0.001
	CON	15.86 ± 3.11	16.11 ± 4.11	G × T = 0.001	-		
Peak knee valgus	NMT	9.19 ± 2.86	4.68 ± 1.68*	G = 0.01	0.98 (0.32 to 1.39) d	0.38 (0.11 to 0.65)	0.03
	VMT	9.96 ± 2.67	5.06 ± 1.43*	T = 0.001	1.01 (0.52 to 1.56) d		0.04
	CON	10.18 ± 2.69	4.86 ± 1.98	G × T = 0.001	-		
Peak vGRF	NMT	3.8 ± 0.5	3.1 ± 0.47*	G = 0.04	1.42 (0.29 to 1.67) d	0.09 (0.02 to 0.36)	0.001
	VMT	3.7 ± 0.6	3.01 ± 0.49*	T = 0.02	1.39 (0.27 to 1.69) d		0.001
	CON	3.7 ± 0.5	3.8 ± 0.48	G × T = 0.001	-		
YBT overall	NMT	83.22 ± 9.89	92.69 ± 9.11*	G = 0.02	1.11 (0.11 to 1.31) d	1.83 (0.76 to 3.26)	0.02
	CG	81.19 ± 10.68	90.86 ± 10.29*	T = 0.03	1.12 (0.13 to 1.32) d		0.03
	CON	82.29 ± 19.68	83.64 ± 9.29	G × T = 0.001	-		
Triple hop test	NMT	446.22 ± 45.11	532.46 ± 50.76*	G = 0.02	1.15 (0.27 to 1.34) d	3.03 (1.22 to 5.11)	0.02
	VMT	448.39 ± 46.73	529.43 ± 51.11*	T = 0.03	1.16 (0.24 to 1.29) d		0.02
	CON	446.76 ± 45.12	445.86 ± 46.29	G × T = 0.001	-		
ACL-RSI scale	NMT	46.12 ± 10.11	75.11 ± 9.11*	G = 0.04	1.62 (0.29 to 1.64) d	1.26 (0.56 to 2.78)	0.001
	VMT	44.73 ± 9.86	73.85 ± 10.12*	T = 0.02	1.63 (0.11 to 1.75) d		0.001
	CON	45.11 ± 10.43	46.11 ± 9.43	G × T = 0.001	-		

NMT: Neuromuscular training program; VMT: Visual-Motor Training; YBT: Y-Balance Test; vGRF: vertical ground reaction force; ACL-RSI: Short form of the short Anterior Cruciate Ligament-Return to Sport after Injury scale; CON; control; G: group; T: time; CI: confidence interval; d: large ES. *Significant differences compared to pre and CON (p < 0.05)

## Discussion

This randomized controlled trial was designed to directly compare the efficacy of 8 weeks of NMT versus VMT on a comprehensive set of outcomes critical to recovery after ACLR. The findings demonstrated that both NMT and VMT interventions elicited significant improvements in landing biomechanics, functional performance, and psychological factors in female athletes with unilateral ACLR. Furthermore, the analysis revealed no statistically significant differences between the two training groups for all post-test measures. This suggests that despite their distinct theoretical foundations, no significant differences were observed between the two interventions in facilitating recovery. This equivalence provides clinicians with compelling evidence for two powerful, yet functionally distinct, rehabilitation pathways. The results of the present study are consistent with those of the previous study [17, 28, 49].

The findings of this study indicated that both NMT and VMT interventions induced profound positive changes in lower limb biomechanics during a single-legged landing task. Athletes in both groups demonstrated significantly increased peak hip, knee, and ankle dorsiflexion, alongside a marked reduction in peak knee valgus and peak GRF_V_ compared to the control group. This collective shift toward a more flexed and aligned landing strategy is critically important from an injury prevention perspective.

The presence of reduced hip, knee, and dorsiflexion, along with increased knee valgus, constitutes a biomechanical profile strongly associated with a heightened risk of both primary and secondary ACL injuries [50]. Therefore, interventions that effectively mitigate these detrimental joint positions are advantageous for patients. The observed reduction in peak GRF_V_ is indicative of a softer landing pattern, a finding that aligns well with previous research on programs incorporating strength and plyometric exercises [51–53]. This reduction in GRFV may be associated with a lower risk of ACL injury [54]; however, it is important to note that knee extension moments were not measured or reported in this study. Additionally, when discussing unilateral loading and kinetic analysis, it is crucial to consider how load distribution and kinetic parameters are equated or analyzed, as highlighted in recent studies comparing unilateral and bilateral movements [55]. The internal knee extension moment is influenced by factors such as increased quadriceps muscle activity, which can lead to greater anterior tibial shear force [56]. Increased knee valgus has been linked to an elevated risk of ACL injuries [50, 57]. Therefore, by promoting landing strategies that reduce both frontal-plane (valgus) and sagittal-plane (extension moment) loads, both NMT and VMT appear to effectively mitigate hazardous knee loading.

Moreover, the observed improvements in joint kinematics and kinetics suggest an enhancement in lower extremity stiffness and dynamic postural control. It is widely accepted that the stiff, less-flexed landing pattern is a key deficit post-ACLR. The significant increases in flexion angles following our interventions suggest that both training modalities successfully enhanced the body's ability to control and dissipate landing forces through a greater range of motion, thereby protecting the passive restraints of the knee [58]. This improvement in fundamental movement quality likely contributed to the concurrent enhancements in functional performance, as evidenced by the significant gains in the triple-hop test and YBT.

The triple hop test, while it reflects power and stability, can sometimes mask underlying biomechanical deficits [59]. The fact that both the NMT and VMT groups displayed improved performance suggests a more robust and genuine functional recovery. Similarly, performance scores on the YBT are a validated tool for identifying bilateral neuromuscular control deficits [60]. Therefore, the significant improvements in the YBT observed in both intervention groups provide strong evidence for the effectiveness of both NMT and VMT in restoring the dynamic stability and sensorimotor integration necessary for athletic performance. These changes can be attributed to the specific characteristics of the interventions. In the VMT group, athletes were often instructed to close their eyes during variable motor training exercises. This approach is hypothesized to enhance proprioceptive training, potentially improving sport-specific performance and cognitive aspects such as visual memory, anticipatory timing of movements, and sensitivity to motion in the central visual field, which collectively may lead to improved attention abilities [28].

In the current study, a statistically significant increase in the ACL-RSI scale score was observed in both the NMT and VMT groups compared to the control group. Both intervention groups demonstrated significant improvements from pre- to post-test assessments. As patients undergo visual obstruction training, they become more attuned to their body’s position and movements, fostering a sense of control and mastery over their physical capabilities, which improves self-efficacy and reduces anxiety related to movement [61]. Additionally, the neurophysiological changes induced by VMT, enhancing sensorimotor processing, contribute to psychological resilience; as individuals experience improved motor control and functional outcomes, their confidence in physical abilities is likely to increase, further mitigating fears associated with movement. Furthermore, VMT promotes better cortical top-down motor control strategies, leading to a more positive body image and self-perception [28]. As patients recognize their improved capabilities, psychological barriers may diminish, facilitating a more positive outlook towards rehabilitation. Thus, while VMT primarily targets physical recovery through enhanced proprioceptive input, its implications for psychological factors warrant further exploration to enrich rehabilitation protocols and support holistic recovery in ACL injury patients [30]. This finding is clinically relevant because during the rehabilitation process, patients exhibiting high levels of kinesiophobia, fear of re-injury, are identified using clinical questionnaires such as the ACL-RSI [41]. Once individuals with elevated fear levels are recognized, targeted interventions, such as goal setting, can be implemented to enhance rehabilitation outcomes [62]. It is also essential to evaluate movement patterns during functional tasks and address any deficits or abnormal movements throughout rehabilitation [63]. These strategies can facilitate a safer return to sport or physical activity and help mitigate the risk of future injuries [64]. Implementing a sport injury risk profile encourages consideration of various factors, including sociocultural influences (e.g., expectations from coaches or teams regarding return-to-sport timelines), mixed psychological states (e.g., fear of re-injury), and the evolving goals of athletes during recovery [62]. Athletes must also navigate the complex neurocognitive and environmental factors associated with returning to sport, including weather conditions, reactions from fans or opponents, and altered decision-making processes in competitive settings [64]. It is well established that neurocognition and emotional states significantly impact adherence to rehabilitation programs, which is a critical factor for successful recovery [62]. Therefore, it is recommended to take into account the myriad psychosocial factors that athletes with ACLR must manage during their rehabilitation journey to optimize recovery outcomes. While prior research has robustly established the efficacy of NMT, evidence for VMT in this population is emerging. Our finding that VMT produces comparable improvements to the gold-standard NMT represents a significant expansion of the evidence-based toolkit available for ACLR rehabilitation. The findings of this study indicated that both NMT and VMT are highly effective rehabilitation strategies.

### Limitations and future scope

This study provides valuable insights; however, several limitations should be acknowledged. First, the use of a convenience sampling method introduces potential selection bias. Consequently, the sample may not adequately represent the broader target population, which limits the external validity and generalizability of the results. Unlike probability sampling, convenience sampling does not allow for random selection of participants; therefore, the findings should be interpreted as preliminary and specific to the accessible population from which the sample was drawn. Future research should employ random or stratified sampling methods to enhance the representativeness of the sample and the applicability of the findings to other settings or populations. Second, the study involved a relatively homogenous sample of female athletes who had undergone hamstring autograft ACLR, which may limit the generalizability of the findings to male athletes, individuals with different graft types, or those with concomitant injuries. Third, the observation period for follow-up after the training intervention was limited; implementing various follow-up durations (e.g., 2-, 4-, or 6-months post-training) could yield a more comprehensive understanding of the long-term effectiveness of both NMT and VMT training. Additionally, while assessor blinding was mentioned, participants undergoing stroboscopic training could not realistically be blinded. Therefore, the potential placebo effect should be addressed in the limitations. Furthermore, the kinematic analysis was conducted using a two-dimensional (2D) approach. This is a methodological limitation, as 2D analysis cannot capture the full complexity of movement mechanics, including three-dimensional kinematic and kinetic variables that could provide deeper insights into joint dynamics and multiplanar control strategies. In addition, this study is limited by the absence of long-term follow-up and retention testing, which restricts the ability to assess the durability of the intervention effects over time. Future research should include these elements to provide a more comprehensive understanding of the long-term outcomes associated with the interventions. In summary, while our findings contribute to the understanding of rehabilitation strategies post-ACLR, these limitations should be considered when interpreting the results and their applicability to broader populations.

## Conclusion

The findings of this study suggest that both NMT and VMT may be effective rehabilitation strategies for improving various outcomes following ACLR. Both programs showed significant improvements in landing biomechanics, functional performance, and psychological factors in female athletes with unilateral ACLR. However, the lack of statistically significant differences between the two training modalities indicates that clinicians have flexibility in selecting a rehabilitation approach based on individual athlete needs, preferences, and available resources. It is important to note that the conclusions drawn may be stronger than the evidence supports, particularly regarding the generalizability of the results. Future research should prioritize mechanism-driven studies and larger randomized controlled trials (RCTs) to further validate these findings and enhance their applicability across diverse athlete populations. Additionally, addressing psychological factors related to fear of re-injury.

## Supplementary Information

Below is the link to the electronic supplementary material.

Supplementary Material 1

## Data Availability

The datasets used and/or analyzed during the current study are available from the corresponding author on reasonable request.
